# Intestinal Colonization with *Tropheryma whipplei*—Clinical and Immunological Implications for HIV Positive Adults in Ghana

**DOI:** 10.3390/microorganisms9081781

**Published:** 2021-08-22

**Authors:** Kirsten Alexandra Eberhardt, Fred Stephen Sarfo, Eva-Maria Klupp, Albert Dompreh, Veronica Di Cristanziano, Edmund Osei Kuffour, Richard Boateng, Betty Norman, Richard Odame Phillips, Martin Aepfelbacher, Torsten Feldt

**Affiliations:** 1 Department of Tropical Medicine, Bernhard Nocht Institute for Tropical Medicine & I. Department of Medicine, University Medical Center Hamburg-Eppendorf, 20359 Hamburg, Germany; 2 Institute for Transfusion Medicine, University Medical Center Hamburg-Eppendorf, 20251 Hamburg, Germany; 3 Department of Medicine, Kwame Nkrumah University of Science and Technology, 00233 Kumasi, Ghana; stephensarfo78@gmail.com (F.S.S.); branorman@yahoo.com (B.N.); phillips@kccr.de (R.O.P.); 4 Department of Medicine, Komfo Anokye Teaching Hospital, 00233 Kumasi, Ghana; 5 Institute of Medical Microbiology, Virology and Hygiene, University Medical Centre Hamburg-Eppendorf, 20251 Hamburg, Germany; e.klupp@uke.de (E.-M.K.); maepfelbacher@uke.de (M.A.); 6 Department of Clinical Microbiology, Komfo Anokye Teaching Hospital, 00233 Kumasi, Ghana; adompreh@gmail.com (A.D.); richardboateng166@gmail.com (R.B.); 7 Institute of Virology, Faculty of Medicine and University Hospital Cologne, University of Cologne, 50935 Cologne, Germany; veronica.di-cristanziano@uk-koeln.de; 8 Laboratory of Retrovirology, The Rockefeller University, New York, NY 10065, USA; eosei@rockefeller.edu; 9 Kumasi Center for Collaborative Research in Tropical Medicine, 00233 Kumasi, Ghana; 10 Clinic of Gastroenterology, Hepatology and Infectious Diseases, University Hospital Düsseldorf, 40225 Düsseldorf, Germany; torsten.feldt@med.uni-duesseldorf.de

**Keywords:** Whipple’s disease, enteric infections, Sub-Sahara, Africa, epidemiology

## Abstract

Background: Recent studies demonstrated higher prevalence rates of *Tropheryma whipplei* (*T. whipplei*) in HIV positive than in HIV negative subjects. However, associations with the immune status in HIV positive participants were conflicting. Methods: For this cross-sectional study, stool samples of 906 HIV positive and 98 HIV negative individuals in Ghana were tested for *T. whipplei*. Additionally, sociodemographic parameters, clinical symptoms, medical drug intake, and laboratory parameters were assessed. Results: The prevalence of *T. whipplei* was 5.85% in HIV positive and 2.04% in HIV negative participants. Within the group of HIV positive participants, the prevalence reached 7.18% in patients without co-trimoxazole prophylaxis, 10.26% in subjects with ART intake, and 12.31% in obese participants. Frequencies of clinical symptoms were not found to be higher in HIV positive *T. whipplei* carriers compared to *T. whipplei* negative participants. Markers of immune activation were lower in patients colonized with *T. whipplei*. Multivariate regression models demonstrated an independent relationship of a high CD4+ T cell count, a low HIV-1 viral load, and an obese body weight with the presence of *T. whipplei*. Conclusions: Among HIV positive individuals, *T. whipplei* colonization was associated with a better immune status but not with clinical consequences. Our data suggest that the withdrawal of co-trimoxazole chemoprophylaxis among people living with HIV on stable cART regimen may inadvertently increase the propensity towards colonization with *T. whipplei*.

## 1. Introduction

*Tropheryma whipplei* (*T. whipplei*) is the causative agent of Whipple’s disease, a rare systemic infectious disease. Classic Whipple’s disease typically involves the gastrointestinal tract and is associated with diverse intra- and extra-intestinal manifestations [1,2,3,4]. However, the detection of *T. whipplei* in asymptomatic carriers indicates that colonization is common and that only a minority of infected individuals ultimately develop Whipple’s disease upon exposure to this soil microbe [5,6].

The prevalence of *T. whipplei* varies considerably across studies, subjects and geographic regions. Detection rates observed in resource-limited settings are generally higher than in more developed countries [7,8,9,10]. Furthermore, children are at higher risk of being colonized than adults [6,8,11]. Differences between sample specimens and applied PCR assays might also contribute to observed variations [12].

Symptomatic cases of Whipple’s diseases are commonly treated with short-term parenteral ceftriaxone or penicillin, followed by oral co-trimoxazole for one year [1]. At the same time, daily co-trimoxazole is recommended for African adults living with HIV, irrespective of antiretroviral treatment, immune status, or disease change [13]. As *T. whipplei* is susceptible to co-trimoxazole, potential long-term prophylaxis has to be accounted for when interpreting prevalence rates in HIV positive African populations.

Studies from Europe and the US reported higher prevalence and abundance rates of *T. whipplei* in people living with HIV (PLHIV) than in HIV negative subjects [14,15,16]. Further, Lozupone et al. observed a decline of this microbe in respiratory samples after initiation of antiretroviral therapy (ART), whereas a longitudinal study in healthy subjects without any antibiotic or antiviral treatment demonstrated a persistence of *T. whipplei* over time [17]. A study from Spain found increased prevalence rates in HIV positive subjects with metabolic syndrome (MetS) compared to PLHIV without MetS [15]. Since long-term antiretroviral treatment increases the chance of developing MetS, these results contrast those from Lozupone et al. to a certain degree [18].

By investigating the prevalence of *T. whipplei* in stool samples of PLHIV in Sub-Saharan Africa, we aim to contribute to the understanding of this pathogen and its clinical relevance in immunocompromised subjects with varying immune status and anti-retroviral and antibiotic treatment experiences.

## 2. Materials and Methods

### 2.1. Study Setting

This cross-sectional study is part of a larger study investigating the prevalence of *Helicobacter pylori* (*H. pylori*) and other gastrointestinal pathogens in HIV positive and negative adults in the Ashanti Region, Ghana [19,20,21]. Between November 2011 and November 2012, consecutive HIV positive patients presenting to the HIV outpatient department of the Komfo Anokye Teaching Hospital, and HIV negative blood donors, were offered participation in the study. All participants gave a written informed consent prior to enrolment. The study was approved by the appropriate ethics committees in Ghana (CHRPE/AP/12/11) and Germany (PV3771). Reporting followed the STROBE (Strengthening the Reporting of Observational Studies in Epidemiology Statement) recommendations (Supplementary File).

### 2.2. Data Collection and Laboratory Methods

Trained study personnel collected demographic and clinical data using a standardized questionnaire. Blood samples were collected, and the analysis of CD4+ T cell count was performed in Ghana using a FACSCalibur flow cytometer (Becton Dickinson, Mountain View, CA, USA). HIV-1 viral load was measured using the Real-Time HIV-1 PCR system (Abbott Diagnostics, Wiesbaden, Germany).

Peripheral blood mononuclear cells (PBMCs) were isolated by centrifugation of heparinized venous blood on a Ficoll/Hypaque (Biocoll Seperating Solution, Biochrom AG, Berlin, Germany) density gradient. Cells were washed in phosphate-buffered saline and resuspended in Roswell Park Memorial Institute 1640 medium (both Gibco Invitrogen, Carlsbad, CA, USA) supplemented with heat-inactivated fetal calf serum (Biochrom AG, Berlin, Germany). PBMCs were cryopreserved and shipped to Germany on liquid nitrogen. Cell surface markers for immune activation were stained as described in our previous work [22]. Flow cytometric data were acquired using the LSRII flow cytometer (BD Biosciences, Heidelberg, Germany) and analyzed using FlowJo version 9.6.2 (Tree Star, San Carlos, CA, USA).

Aliquots of native stool samples were freshly frozen and stored at −80 °C before being transported to Germany on dry ice. Nucleic acids were extracted applying the QiaAMP DNA Stool Mini kit (Qiagen, Hilden, Germany). Real-time PCR for *T. whipplei* was performed as previously published [23,24]. Briefly, primer and probe for target 1: TW27-F TGTTTTGTACTGCTTGTAACAGGATCT; TW182-R TCCTGCTCTATCCCTCCTATCAT, TX27182P, FAMAGAGATACATTTGTGTTAGTTGTTACA-BHQ-1; and for target 2: TW13-F TGAGTGATGGTAGTCTGAGAGATATGT; TW163-R TCCATAACAAAGACAACAACCAATC, TW13163P, FAMAGAAGAAGATGTTACGGGTTG-BHQ1 were used. Both PCRs (TW27-182 and TW13-163) were performed in a 25-μL total volume using Roche LightCycler 480 II (Roche, Mannheim, Germany), the Quantifast Pathogen PCR Kit (Qiagen) and 5 μL of nucleic acid eluate. Only samples positive for both targets (TW27-182 and TW13-163, both with Ct values ≤ 37) were defined as *T. whipplei* positive.

### 2.3. Statistical Analyses

Continuous variables were expressed as median (interquartile range, IQR) or mean ± standard deviation (SD) and compared using the Wilcoxon rank sum test or the unpaired Student’s t-test. Categorical variables were compared using either the χ^2^ test or the Fisher exact test, as appropriate. Potential associations of independent parameters with the *T. whipplei* status were assessed by applying multivariate logistic regression models adjusting for demographic and treatment-related parameters using the ‘forestmodel’ package in R (version 4.0.5, R Foundation for Statistical Computing, Vienna, Austria). To account for moderation, models were additionally run with included interaction terms. Only available data were considered for analysis without application of imputation methods in case of missing data points. Two-sided *p*-values were presented, and an α of 0.05 was determined as the cutoff for significance.

## 3. Results

**Figure 1:**
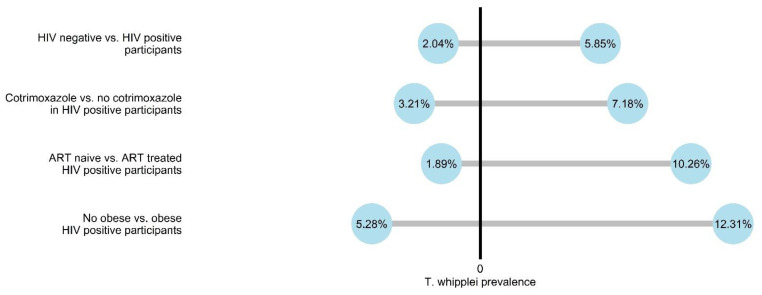
Prevalence of *T. whipplei* according to cohort characteristics.

A total of 1095 HIV positive individuals and 107 HIV negative blood donors were recruited for this study. Stool samples for *T. whipplei* testing were available for 906 (82.7%) HIV positive and 98 (91.6%) HIV negative individuals. The prevalence of *T. whipplei* was 5.85% (*n* = 53) in HIV positive participants and 2.04% (*n* = 2, *p* = 0.158) in HIV negative participants (Figure 1). Within the group of HIV positive participants, the prevalence varied depending on subgrouping and reached 7.18% in patients without co-trimoxazole prophylaxis (vs. 3.21% in those with co-trimoxazole intake, *p* = 0.021), 10.26% in subjects with ART intake (vs. 1.89% in ART-naïve patients, *p* < 0.001) and 12.31% in obese HIV positive participants (vs. 5.28% in non-obese subjects, *p* = 0.046).

HIV positive participants with present or absent *T. whipplei* were not different with regards to age, sex, or any of the recorded socioeconomic factors (Table 1). Furthermore, frequencies of clinical symptoms were not found to be higher in HIV positive *T. whipplei* carriers compared to *T. whipplei* negative participants.

**Figure 2:**
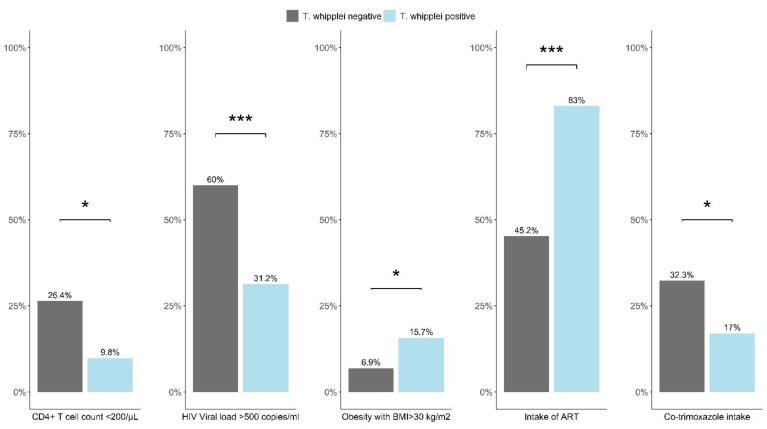
Laboratory, medical, and treatment-related parameters according to *T. whipplei* status among HIV positive participants. BMI—Body mass index; ART—antiretroviral therapy; *—*p* < 0.05; ***—*p* < 0.001.

HIV positive participants who were co-infected with *T. whipplei* were more frequently receiving antiretroviral therapy and less common co-trimoxazole as antibiotic prophylaxis (83.02%, vs. 45.19%, *p* < 0.001, and 16.98% vs. 32.26%, *p* = 0.030, Figure 2). Subsequently, a smaller proportion of *T. whipplei* carriers had viral loads above 500 copies/mL or CD4+ T cell counts lower than 200 cells/µL detected (31.25% vs. 60.00%, *p* < 0.001, and 9.80% vs. 26.36%, *p* = 0.013, respectively). Furthermore, *T. whipplei* positive individuals presented more than twice as often with an obese body weight (Body mass index [BMI] > 30 kg/m^2^) than those tested negative for *T. whipplei* (15.69% vs. 6.88%, *p* = 0.040).

In addition to lower HIV viral loads and higher CD4+ T cell counts (1.6 [0.0–3.3 IQR] vs. 4.0 [1.6–5.3 IQR], *p* < 0.001, and 525.0 [371.0–654.5 IQR] vs. 396.0 [184.0–632.5 IQR], *p* = 0.007, respectively), HIV positive individuals with *T. whipplei* carriage had a higher CD4+/CD8+ T cell ratio (0.5 [0.4–0.8 IQR] vs. 0.4 [0.2–0.7 IQR], *p* = 0.020) and a lower expression of HLA-DR+CD38+ on CD4+ T lymphocytes (11.2% [7.3–19.7 IQR] vs. 18.4% [10.4–32.8 IQR], *p* = 0.009) and CD8+ T lymphocytes (29.1% [22.7–40.5 IQR] vs. 42.3% [27.5–55.7 IQR], *p* = 0.006), as markers of decreased immune activation (Table 2). Furthermore, individuals with *T. whipplei* had a higher proportion of CD57+, as a marker for terminal differentiation on CD8+ T lymphocytes (54.6% [44.8–68.5 IQR] vs. 49.0% [38.9–60.3 IQR], *p* = 0.049).

**Figure 3:**
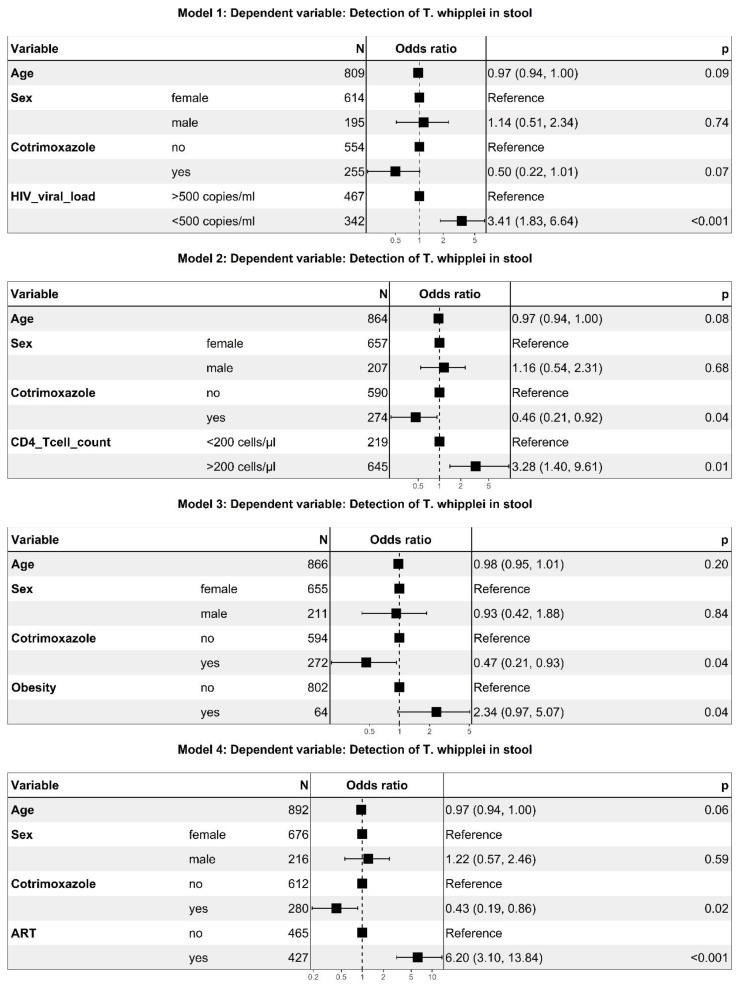
Factors associated with *T. whipplei* carriage in HIV positive participants. Multiple logistic regression models displaying odds ratios (95% confidence intervals) and adjusted *p*-values of independent variables. Obesity defined as BMI > 30 kg/m². ART—antiretroviral therapy.

In order to determine if virological and immunological parameters were independently associated with the presence of *T. whipplei*, we adjusted for the intake of co-trimoxazole, sex, and age in individual multivariate logistic regression models. Figure 3 demonstrates that both an HIV viral load below 500 copies/mL as well as a CD4+ T cell count above 200 cells/µL were associated with a detection of *T. whipplei* in stool samples, independently from the intake of co-trimoxazole (3.41 OR [1.83–6.64, 95%CI], and 3.28 OR [1.40–9.61, 95%CI], respectively). The third model presented indicates that obesity, defined as a BMI above 30 kg/m², also is correlated with a higher probability of *T. whipplei* carriage (2.34 OR [0.97–5.07, 95%CI]). Since viral load, CD4+ T cell count, and body weight are affected by the intake and effectiveness of antiretroviral treatment, we added a fourth model demonstrating that ART treatment independently correlates with the presence of *T. whipplei* in stool samples after correction for co-trimoxazole and demographic parameters (6.20 OR [3.10–13.84, 95%CI]). An alternative interactive logistic regression model revealed no significant interaction effect between co-trimoxazole and ART intake.

## 4. Discussion

To the best of our knowledge, this is the first study investigating the prevalence, risk factors, clinical relevance, and immunological implications of intestinal *T. whipplei* colonization in a large cohort of HIV positive individuals in Sub-Saharan Africa and one of only few studies on *T. whipplei* in HIV infected subjects in general.

The prevalence of *T. whipplei* found in our study population was lower than in other studies from Sub-Saharan Africa [5,6,7]. One cause might be that we included only adult subjects and the majority of those were female (>75%), with both characteristics being associated with lower rates of *T. whipplei* carriage [6,8]. Another aspect is that the cohort studied in our work was recruited at the center of Kumasi, Ghana, a city of 3.3 million inhabitants. The finding that more than half of the participants indicated to have access to tap water and almost all had electricity available within their households suggests that risk factors for acquiring *T. whipplei* in urban areas might differ from those in rural settings.

In agreement with a study on PLHIV in Europe, there was no accumulation of acute or chronic symptoms in subjects carrying *T. whipplei* in our cohort from Sub-Saharan Africa [15]. Likewise, Qin et al. reported no impairment of pulmonary function in PLHIV with *T. whipplei* positive bronchoalveolar lavage samples [14]. Our findings are also in line with a recent work from Ghana in which *T. whipplei* was found equally in symptomatic pediatric cases and controls [25]. Despite earlier research indicating that individuals might develop mild diarrhea when infected, Feurle et al. concluded in their study of symptomatic patients from different origins that *T. whipplei* was associated with the presence of other stool pathogens, but that an independent causative role of this sole microbe in diarrhea appeared unlikely [26,27,28].

Co-trimoxazole is recognized for its activity against *T. whipplei* [1]. Since more than 30% of our HIV positive study population was receiving co-trimoxazole as long-term prophylaxis with a subsequent reduced colonization rate (7.18% vs. 3.21%, *p* = 0.021), we adjusted for the intake of co-trimoxazole in further multivariate models.

In line with earlier studies, the prevalence of *T. whipplei* was higher in ART-treated HIV positive subjects than in the HIV negative control group [15,16]. While Lozupone et al. were not able to detect differences in *T. whipplei* rates between ART-treated and ART-naïve individuals, possibly due to small sample sizes, we found this pathogen to be significantly more prevalent in patients receiving ART (10.26% vs. 1.89%, *p* < 0.001) [16]. Also, after adjustment for confounders, a high CD4+ T cell count and low HIV-1 viral load as indicators of an effective ART treatment remained significantly associated with a positive *T. whipplei* status, independent from co-trimoxazole prophylaxis. In addition to this, markers of immune activation were found to be decreased in *T. whipplei* carriers in our cohort. This finding is in agreement with a study on *T. whipplei* colonization of the lung in PLHIV, where *T. whipplei* positivity was not associated with increased systemic or local inflammation [14]. Indeed, Moos and Schneider related an absence of an inflammatory response against *T. whipplei* to the establishment of a chronic infection, which might in part explain the higher prevalence in individuals receiving anti-inflammatory antiretroviral therapy [29]. Obesity was also observed more often in *T. whipplei* positive compared to negative participants (12.31% vs. 5.28%, *p* = 0.046). Weight loss is a common finding in advanced HIV disease, and an increase in BMI after initiation of first line ART has been described [30]. A similar observation of increased *T. whipplei* prevalence rates in HIV positive subjects with MetS was made by Garcia-Alvarez et al. [15].

The underlying mechanisms for the observed association of ART intake and parameters indicating efficacy of ART, in particular a low HIV viral load and a high CD4+ cell count, are not obvious. Interestingly, authors of a longitudinal observation of 29 HIV positive subjects found that ART initiation resulted in a decrease of the relative abundance of *T. whipplei* within individuals, which in a way contrasts our result of lower *T. whipplei* rates in ART-naïve subjects and associations of favorable immune parameters with *T. whipplei* carriage [16]. However, Lozupone et al. were testing respiratory specimen for the presence of *T. whipplei* in that study, whereas we investigated stool samples. Although a longitudinal study in a cohort of healthy subjects suggested that individuals with *T. whipplei* in the lung were also likely to have this identical microbe detected in the stomach, it might be presumed that immunologic control is differently affected by HIV (and related treatment) in the respiratory and gastrointestinal tract [17].

One limitation of our study is that we did not record dynamics of immune parameters (e.g., lowest individual CD4+ T cell counts), or the duration of co-trimoxazole prophylaxis. A severely impaired immune status is associated with more frequent infections, clinical complaints, and thus use of antibiotics, with the possible effect of inadvertent *T. whipplei* eradication. Furthermore, there is likely a correlation between the immune status at the time of ART initiation and the administration of co-trimoxazole prophylaxis, which cannot be further explored due to this data gap. Other limitations to mention include the fact that we only screened for *T. whipplei* in stool samples. Although an earlier study by Qin et al. found that multi-site colonization in healthy subjects was common, it would be interesting to investigate if this could be different in advanced HIV disease and if *T. whipplei* is replaced or overgrown by other bacteria in the gastrointestinal, but not in the respiratory tract. A recent evaluation determined the PCR applied in our study to be of high quality (85%), but due to different methods used for the detection of *T. whipplei*, results have to be compared across studies with caution [12]. Because of the cross-sectional design of our study, causal inferences cannot be drawn. A longitudinal study could contribute to the controversial discussion on the impact of HIV disease progression and reversal of immunodeficiency by ART on the prevalence and clinical effects of *T. whipplei* in PLHIV in Africa.

In conclusion, *T. whipplei* colonization was more common in this large cohort of HIV infected individuals in West Africa, compared to HIV negative individuals, but not associated with clinical consequences. Among HIV positive individuals, the intake of ART and not being on co-trimoxazole prophylaxis were strongly associated with *T. whipplei* colonization. Our data suggest that the withdrawal of co-trimoxazole chemoprophylaxis among people living with HIV on stable cART regimen may inadvertently increase the propensity towards colonization with *T. whipplei*. Longitudinal studies are needed to investigate causality and underlying mechanisms.

## Figures and Tables

**Table 1:** Demographical and socioeconomic parameters, and clinical symptoms in HIV infected individuals according to *T. whipplei* status.

	Variable	HIV Positive *T. whipplei* Negative, *n* = 853 (94.15%)	HIV Positive *T. whipplei* Positive, *n* = 53 (5.85%)	*p*-Value
Demographics	Age in years ± SD	40.5 ± 9.5	38.5 ± 7.9	0.127
	Female, n (%)	643 (75.38)	42 (79.25)	0.638
Socioeconomic parameters	Access to tap water, n (%)	449 (52.64)	29 (54.72)	0.879
	Electricity in household, n (%)	792 (92.85)	49 (92.45)	0.787
	Television in household, n (%)	689 (80.77)	42 (79.25)	0.925
	Refrigerator in household, n (%)	603 (70.69)	34 (64.15)	0.392
	Owning a car, n (%)	80 (9.38)	4 (7.55)	0.810
Clinical symptoms during the last six months	Any acute or chronic cough, n (%)	100 (11.74)	3 (5.66)	0.262
	Any acute or chronic gastrointestinal symptoms, n (%)	105 (12.32)	4 (7.55)	0.387
	Any acute or chronic fever, n (%)	84 (9.86)	2 (3.77)	0.222
	Weigh loss during last six months, n (%)	201 (23.59)	6 (11.32)	0.058
	Any acute or chronic symptoms of the above, n (%)	265 (31.10)	10 (18.87)	0.084

SD—standard deviation.

**Table 2:** Virological and immunological parameters according to *T. whipplei* status.

Variable	HIV Positive *T. whipplei* Negative, Median (IQR)	HIV Positive *T. whipplei* Positive, Median (IQR)	*p*-Value
Viral load, log10 copies/ml	4.0 (1.6–5.3)	1.6 (0.0–3.3)	<0.001
CD4+ T-cell count/µL	396.0 (184.0–632.5)	525.0 (371.0–654.5)	0.007
CD8+ T-cell count/µL	960.0 (637.0–1381.0)	1034.0 (588.5–1220.0)	0.763
CD4+/CD8+ T-cell ratio	0.4 (0.2–0.7)	0.5 (0.4–0.8)	0.020
HLA-DR+CD38+CD4+ (%)	18.4 (10.4–32.8)	11.2 (7.3–19.7)	0.009
HLA-DR+CD38+CD8+ (%)	42.3 (27.5–55.7)	29.1 (22.7–40.5)	0.006
CD57+CD4+ (%)	16.4 (9.4–27.6)	13.9 (9.8–26.2)	0.788
CD57+CD8+ (%)	49.0 (38.9–60.3)	54.6 (44.8–68.5)	0.046
PD-1+CD4+ (%)	34.5 (23.8–49.5)	32.8 (22.3–46.3)	0.602
PD-1+CD8+ (%)	31.6 (19.9–43.6)	20.7 (14.9–40.3)	0.074
Ki67+CD4+ (%)	13.0 (6.9–27.2)	9.3 (7.9–11.6)	0.076
Ki67+CD8+ (%)	10.2 (6.0–17.5)	8.4 (6.2–16.8)	0.834

IQR—Interquartile range.

## Data Availability

All relevant data are provided in the manuscript. Raw data can be made available upon reasonable request. Reporting of study methods and results followed the STROBE (Strengthening the Reporting of Observational Studies in Epidemiology Statement) recommendations (Supplementary File).

## Supplementary Materials

The following are available online at https://www.mdpi.com/article/10.3390/microorganisms9081781/s1, STROBE Statement—Checklist of items that should be included in reports of cross-sectional studies.
